# Sesamol Inhibited Ultraviolet Radiation-Induced Hyperpigmentation and Damage in C57BL/6 Mouse Skin

**DOI:** 10.3390/antiox8070207

**Published:** 2019-07-05

**Authors:** Ya-Jhen You, Po-Yuan Wu, Yi-Jung Liu, Chien-Wei Hou, Chin-Sheng Wu, Kuo-Ching Wen, Chien-Yih Lin, Hsiu-Mei Chiang

**Affiliations:** 1 Department of Cosmeceutics, China Medical University, Taichung 40402, Taiwan; 2 Department of Dermatology, China Medical University Hospital, Taichung 40402, Taiwan; 3 School of Medicine, China Medical University, Taichung 40402, Taiwan; 4 Ph.D Program for Biotechnology Industry, China Medical University, Taichung 40402, Taiwan; 5 Department of Biotechnology and Pharmaceutical Technology, Yuanpei University of Medical Technology, Hsinchu 30015, Taiwan; 6 Department of Biotechnology, Asia University, Taichung 41354, Taiwan

**Keywords:** sesamol, melanogenesis, tyrosinase, tyrosinase-related protein-1, microphthalmia-associated transcription factor

## Abstract

Melanin is synthesized through a series of oxidative reactions initiated with tyrosine and catalyzed by melanogenesis-related proteins such as tyrosinase, tyrosinase-related protein-1 (TRP-1), dopachrome tautomerase (TRP-2), and microphthalmia-associated transcription factor (MITF). Our previous study demonstrated that sesamol inhibited melanin synthesis through the inhibition of the melanocortin 1 receptor (MC1R)/MITF/tyrosinase pathway in B16F10 cells. In this study, sesamol was applied to C57BL/6 mouse skin to understand its activity with respect to skin pigmentation. The results indicated that ultraviolet (UV) B-induced hyperpigmentation in the C57BL/6 mouse skin was significantly reduced by topical application of sesamol for 4 weeks. Sesamol reduced the melanin index and melanin content of the skin. In addition, sesamol elevated the brightness (L* value) of the skin. Sesamol also reduced UVB-induced hyperplasia of epidermis and collagen degradation in dermis. In immunohistochemical staining, topical application of sesamol reduced UVB-induced tyrosinase, TRP-1, TRP-2, and MITF expression in the epidermis of the skin. These results demonstrated that sesamol is a potent depigmenting agent in the animal model.

## 1. Introduction

Melanin is synthesized in melanocytes, matured and stored in melanosomes, after which it is transferred through dendrites to keratinocytes, which determine the color of skin. Melanin synthesis is regulated by genes, proteins, and enzymes, namely tyrosinase, microphthalmia-associated transcription factor (MITF), tyrosinase-related protein-1 (TRP-1; 5,6-dihydroxyindole-2-carboxylic acid oxidase), and dopachrome tautomerase (TRP-2) [1,2]. Keratinocytes secrete factors or hormones that bind to specific receptors on melanocytes to initiate melanin synthesis, which involves the stimulation of the secretion of α-melanocyte-stimulating hormones (α-MSHs) that bind to the melanocortin 1 receptor (MC1R) and the activation of cyclic adenylate, which transfers adenosine triphosphate to cyclic adenosine monophosphate (cAMP). Activation of cAMP increases MITF expression to upregulate tyrosinase, TRP-1, and TRP-2 expression, promoting melanin synthesis in melanocytes [3,4].

The types and amounts of melanin are influenced by various extrinsic and intrinsic factors, chiefly hormones, inflammation, and ultraviolet (UV) irradiation. Exposure to sunlight may cause an abnormal increase in the generation of reactive oxygen species (ROS), which may induce skin damage, including collagen degradation in the dermis and hyperplasia of the epidermis [5]. The initiation of melanogenesis may protect the skin from the deleterious effects of UV irradiation and environmental pollutants [6]. However, the excess accumulation of melanin not only causes aesthetic problems but also induces pigmentation disorders [7,8]. Hydroquinone is a skin whitening agent; however, its dermal application has induced slight-to-severe irritation in rats and humans [9,10]. Retinoic acid may cause developmental toxicity. Researchers have sought to identify safe skin whitening agents for the inhibition of hyperpigmentation in the skin [11,12]. Natural products or agents with antioxidant and tyrosinase-inhibiting activities are safer candidates for preventing hyperpigmentation [13,14,15,16].

Sesamol is an active component in sesame seeds. Sesame seeds are a common food in Asian countries. A study reported that sesamol has potent antioxidant and free radical scavenging activities [17,18]. In addition, sesamol exhibited anti-inflammatory activity in another study [19]. A study reported that sesamol decreased UVB-irradiation-induced cytotoxicity, intracellular ROS generation, lipid peroxidation, oxidative DNA damage, and apoptotic morphological changes in human fibroblasts [20]. It was also reported that sesamol inhibited melanin biosynthesis through decreasing the expression of tyrosinase, TRP-1, TRP-2, MITF, and MC1R in melan-a cells [21]. In our previous study, sesamol was found to inhibit melanin synthesis through the regulation of cAMP/protein kinase A (cAMP/PKA), mitogen-activated protein kinase kinase (MEK)/extracellular signal-regulated kinases (ERK), protein kinase B (AKT)/glycogen synthase kinase 3 beta (GSK3β)/CREB, TRP-1, and MITF in B16F10 cells [22]. In addition, sesamol inhibited the activity and protein expression of tyrosinase in melanocytes [22]. This study further investigated the antimelanogenic activity of sesamol in C57BL/6 mice.

## 2. Materials and Methods

### 2.1. Chemicals and Materials

Sesamol, arbutin, and polyethylene glycol (PEG)-400 were obtained from Sigma-Aldrich Chemical Co. (St. Louis, MO, USA). Monobasic potassium phosphate (KH_2_PO_4_) and monobasic sodium phosphate (NaH_2_PO_4_) were obtained from USB Corporation (Cleveland, OH, USA). Ethyl alcohol was purchased from Taiwan Sugar Company (Tainan, Taiwan). Antibodies recognizing TRP-1 and tyrosinase were obtained from Santa Cruz Biotechnology (Santa Cruz, CA, USA). Fontana-Masson (Melanin Stain, Abcam Cambridge, MA, USA) and an antibody recognizing MITF were obtained from Abcam (Cambridge, MA, USA). All other chemicals and reagents used in this study were of high-quality grade and were commercially obtainable.

### 2.2. Preparation of Samples

Sesamol was dissolved in PEG-400 to prepare 1% and 3% sesamol solutions. Arbutin was also dissolved in PEG-400 to prepare a 3% arbutin solution. PEG-400 was used as the vehicle.

### 2.3. Animals and Experimental Design

#### Experimental Animals

The animal experimental protocols were adopted according to the recommendations of the Care and Use of Laboratory Animals and were approved by the Institutional Animal Use and Care Committee of China Medical University (Protocol No.: 104-192-N). Four-week-old female C57BL/6 mice from the National Laboratory Animal Center (Taipei, Taiwan) were obtained for this study. The mice were maintained in the following conditions: temperature, 22 ± 2 °C; relative humidity, 50% ± 10%; and light–dark cycle, 12 h. During this period, a standard laboratory diet and water were provided ad libitum.

### 2.4. UVB Irradiation and Sesamol Treatment

**Figure 1:**
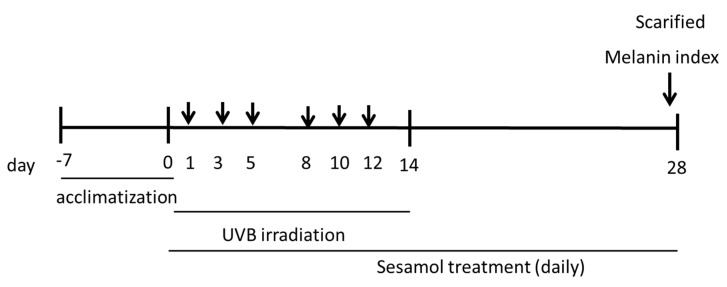
Implementation process of animal experimentation.

After 1 week of acclimatization, the mice were randomly divided into the following five groups: control, UVB-irradiated and vehicle (PEG-400)-treated, UVB-irradiated and 3% arbutin-treated, UVB-irradiated and 1% sesamol–treated, UVB-irradiated and 3% sesamol–treated groups. Each group consisted of six mice. This study used UV light (broadband with peak emission at 302 nm, CL-1000 M, UVP, USA). The mice were exposed to 180 mJ/cm^2^ of UVB irradiation three times a week (on days 1, 3, and 5) for 2 weeks according to previous methods with slight modification [23]. Vehicle-treated UVB-irradiated mice were topically administered PEG on the ear skin daily, and sesamol-treated mice were topically administered 1% or 3% sesamol on the ear skin daily. Sesamol was applied on the skin after UVB irradiation. Control mice did not receive any treatment. Details of the animal experimental process are presented in Figure 1.

### 2.5. Measurement of Melanin Index and L* Value of Mouse Skin

The melanin index of the dorsal skin of the mouse ear was measured using the Mexameter^®^ MX18 of the Multi Skin Test Center (Courage + Khazaka Electronic GmbH, Cologne, Germany). The brightness of the skin (denoted as L*) was measured using a spectrocolorimeter (SCM-104/108, Ruyico Technology Corporation, Taipei, Taiwan).

### 2.6. Preparation of Skin Specimens and H&E Staining

The mice were euthanized through excess carbon dioxide inhalation at the end of the experiment. The mice were macroscopically observed, and their ears were excised, fixed in 10% formaldehyde, and embedded in paraffin. The skin slides were stained with H&E. Collagen in the skin sections was stained with Masson trichrome and was examined under a microscope, as previously described [24,25].

### 2.7. Immunohistochemical Staining

Immunohistochemical staining has been proven to be one of the most powerful techniques in the characterization of the levels and locations of protein in the tissue or organs [26,27]. The skin sections were stained with the monoclonal antimouse antibodies, including tyrosinase, TRP-1, TRP-2, and MITF. The skin sections were examined by microscopy, and the protein expression was determined using Image J software (National Institutes of Health, Bethesda, MD, USA).

### 2.8. Statistical Analyses

Values are expressed as mean ± standard deviation. Student’s *t-*test or analysis of variance was used to analyze differences among all groups; subsequently, Scheffe’s test was used as the post hoc test. *p* values < 0.05 indicated statistical significance.

## 3. Results

### 3.1. Body Weight of Animals

**Figure 2:**
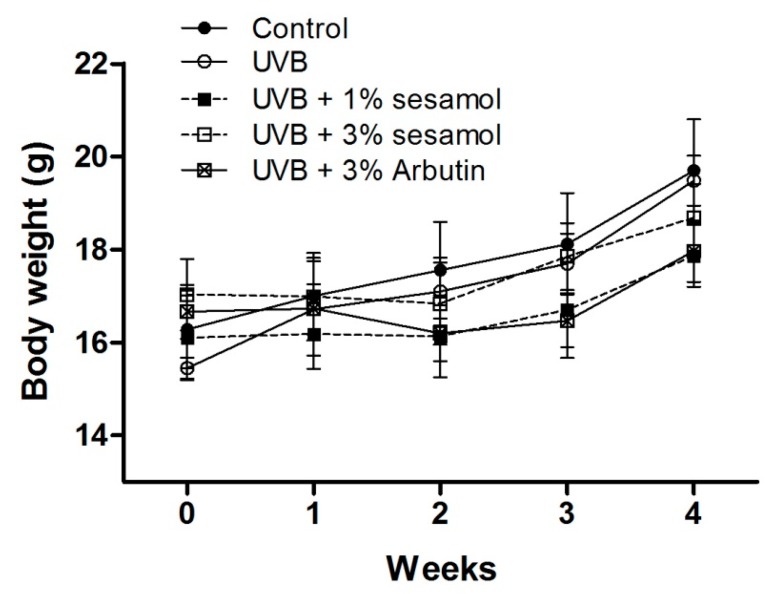
Body weight variation of C57BL/6 mice receiving topical application of sesamol during the 4-week study period.

The body weights of the mice are presented in Figure 2. The body weights were not significantly different in each group after the 4-week experiment.

### 3.2. Melanin Index

**Figure 3:**
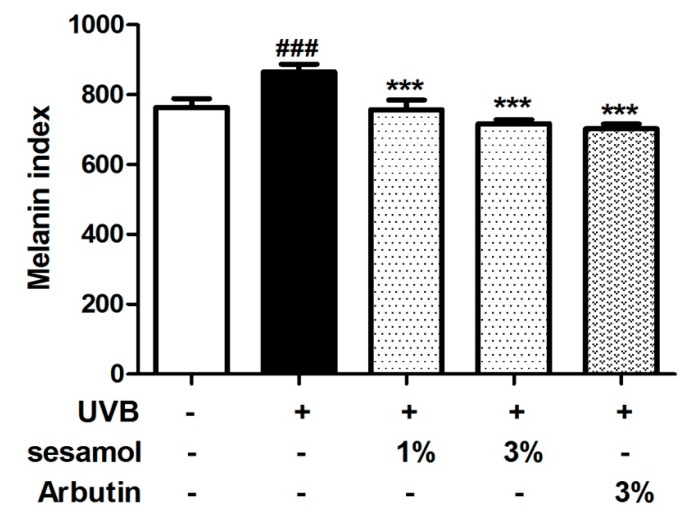
Effect of sesamol on the melanin index of C57BL/6 mouse ear skin post UVB irradiation in the fourth week. UVB irradiation increased the melanin index, whereas topical application of sesamol decreased the melanin index. (Significant difference versus control: ###, *p* < 0.001. Significant difference versus UVB-irradiated group: ***, *p* < 0.001.).

The melanin index of C57BL/6 mice in the control group was 763.5 ± 25.0, which increased to 865.4 ± 21.9 after UVB exposure for 4 weeks (Figure 3). However, 1% and 3% sesamol treatment for 4 weeks reduced the melanin index to 757.4 ± 27.0 and 717.4 ± 11.5, respectively. The effect of 3% sesamol was similar to that of the same concentration of arbutin (Figure 3). The results suggested that sesamol significantly reduced UVB-induced hyperpigmentation in the mouse skin.

### 3.3. L* Value and Hypopigmentation of the Mouse Ear from Sesamol Treatment

**Figure 4:**
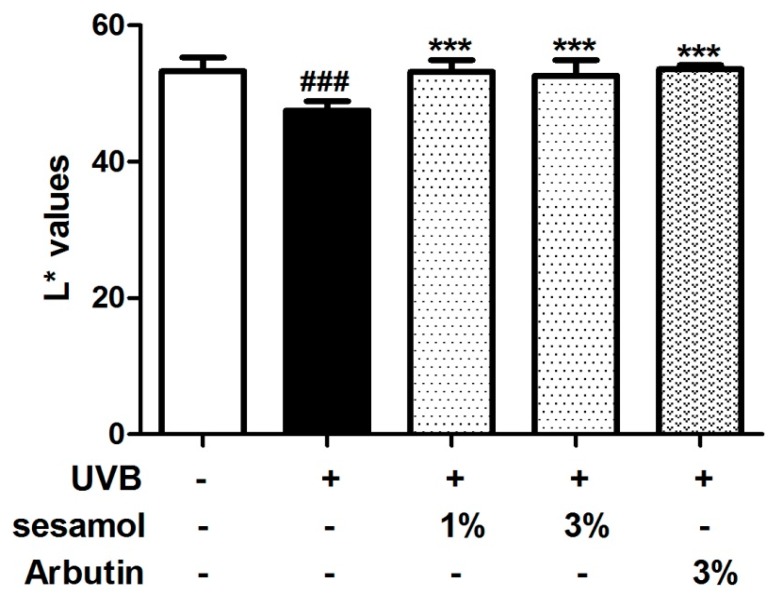
Effect of sesamol on L* values (brightness) of C57BL/6 mouse ear skin after UVB irradiation in the fourth week. UVB irradiation decreased L* values, whereas topical application of sesamol increased L* values. (Significant difference versus control: ###, *p* < 0.001. Significant difference versus UVB treated group: ***, *p* < 0.001.).

**Figure 5:**
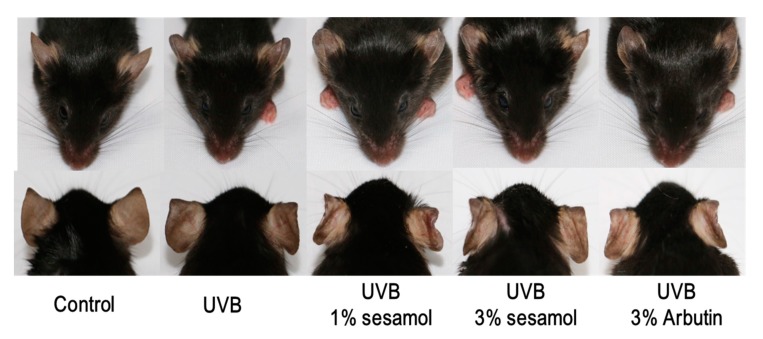
Photographs illustrate the skin lightening effect of sesamol after topical application for 4 weeks on UVB-irradiation-induced hyperpigmentation in C57BL/6 mice.

The L* value represents brightness, and the higher the L* value is, the brighter the skin is. As illustrated in Figure 4, the L* value of C57BL/6 mice in the control group was initially 53.3 ± 2.1 and decreased to 47.5 ± 1.3 after UVB exposure for 4 weeks. However, the L* value increased to 53.2 ± 1.8 and 52.6 ± 2.3 after the 1% and 3% sesamol treatment for 4 weeks, respectively. The results suggest that sesamol exhibits hypopigmentation activity. As indicated in Figure 5, UV exposure induced hyperpigmentation of the ear skin of the mice, whereas sesamol treatment exhibited depigmentation activity. In addition, the effect of sesamol on depigmentation was similar to that of arbutin.

### 3.4. Histochemical Staining

#### 3.4.1. Melanin Content Determined Through Fontana-Masson Staining

**Figure 6:**
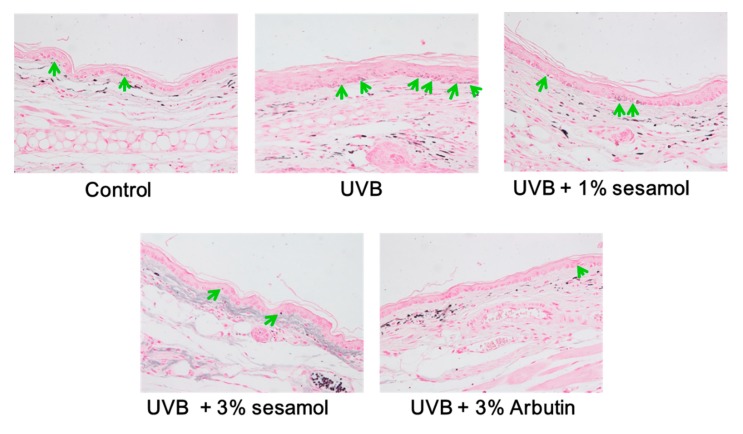

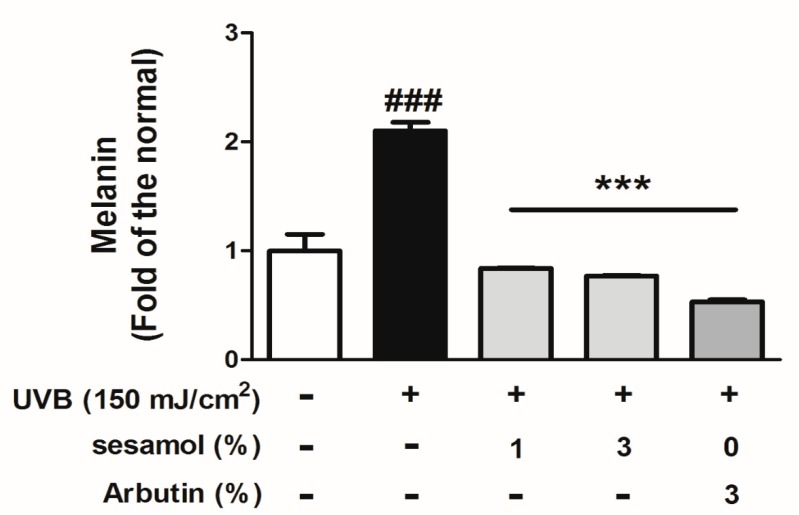
Effect of sesamol on UVB-irradiation-induced hyperpigmentation in C57BL/6 mice ear skin. Melanin pigments (as indicated by the arrows) were stained black by Fontana-Masson staining (400X). Topical application of sesamol reduced melanin pigments. (Significant difference versus control: ###, *p* < 0.001. Significant difference versus UVB treated group: ***, *p* < 0.001.).

Melanin was stained through Fontana-Masson staining, and the results are presented in Figure 6. UVB induced melanin synthesis (as indicated by the arrows), whereas sesamol treatment decreased melanin content in the epidermis of the mouse ear. The effect of sesamol on anti-melanogenesis was similar to that of arbutin.

#### 3.4.2. Thickness of the Epidermis

**Figure 7:**
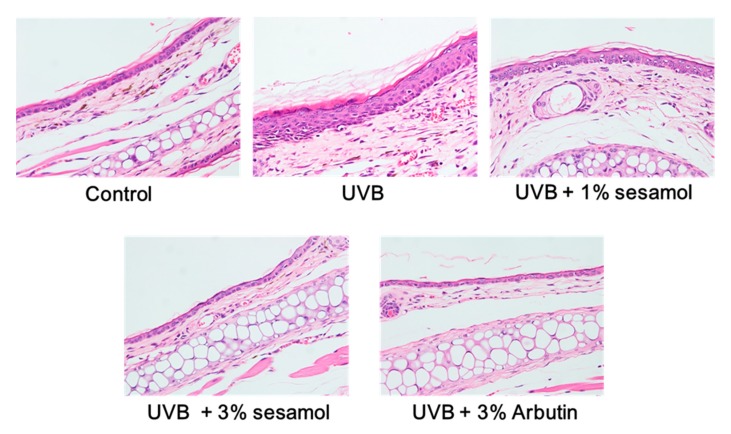

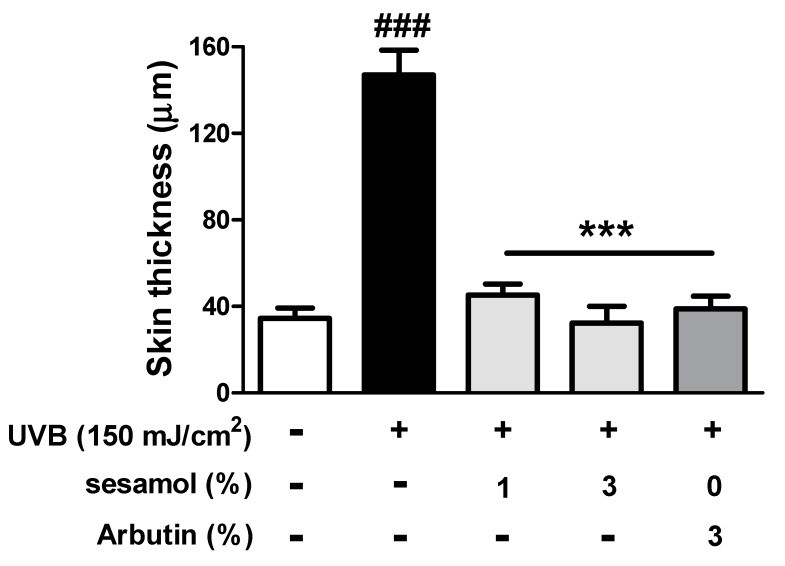
Effect of sesamol on UVB-irradiation-induced histopathological changes in C57BL/6 mouse ear skin. Tissue morphology was visualized through hematoxylin and eosin (H&E) staining (400X). UVB irradiation caused hyperplasia in the skin, whereas topical application of sesamol once a day to the ear skin reduced the thickness of the epidermis. (Significant difference versus control: ###, *p* < 0.001. Significant difference versus UVB treated group: ***, *p* < 0.001.).

Exposure to UV light will induce hyperplasia in the skin. According to the results of hematoxylin and eosin (H&E) staining, UVB caused epidermal hyperplasia, but sesamol reversed this effect (Figure 7). Sesamol may protect the skin from UVB-induced epidermal thickening and skin damage.

#### 3.4.3. Collagen in the Dermis Determined Using Masson’s Trichrome

**Figure 8:**
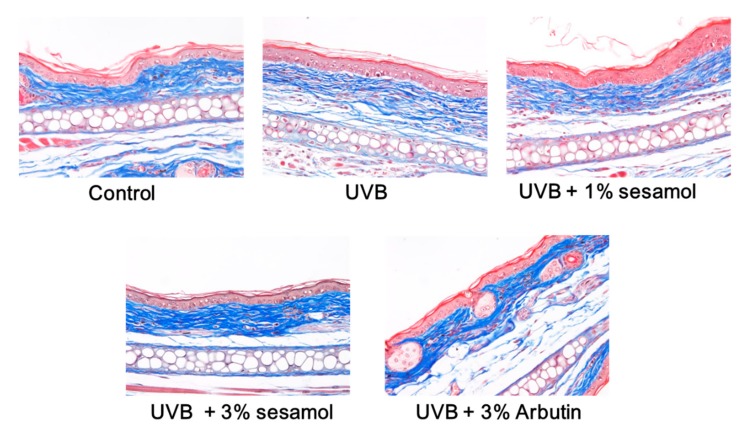

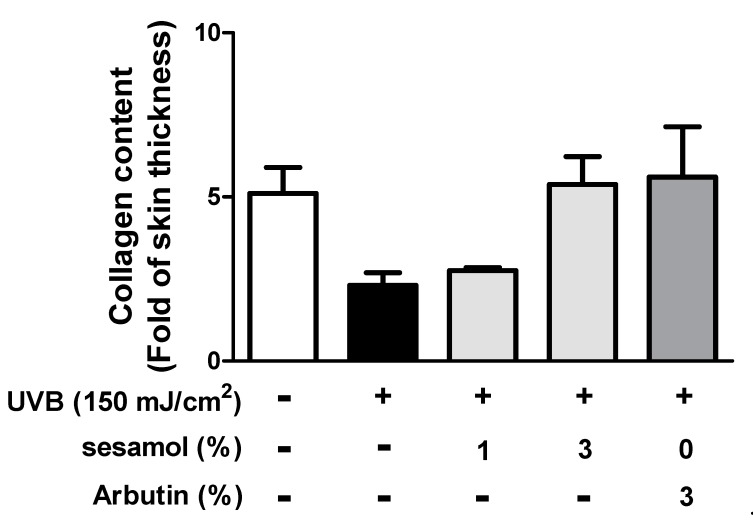
Light micrographs of histological sections stained with Masson’s trichrome in UVB-irradiated C57BL/6 mice that received topical application of sesamol once a day to the ear skin. The collagen content decreased in the UVB-irradiated group, whereas topical application of sesamol once a day to the ear skin restored the collagen level.

Collagen in the dermis was stained blue by Masson’s trichrome. In Figure 8, it is evident that the content and density of collagen in the dermis of the ear were reduced after UVB exposure, whereas topical application of sesamol restored the collagen level in the ear dermis, while the difference was not statistically significant.

### 3.5. Sesamol Inhibited Melanin-Synthesis-Related Protein Levels in UVB-Exposed Mouse Skin

**Figure 9:**
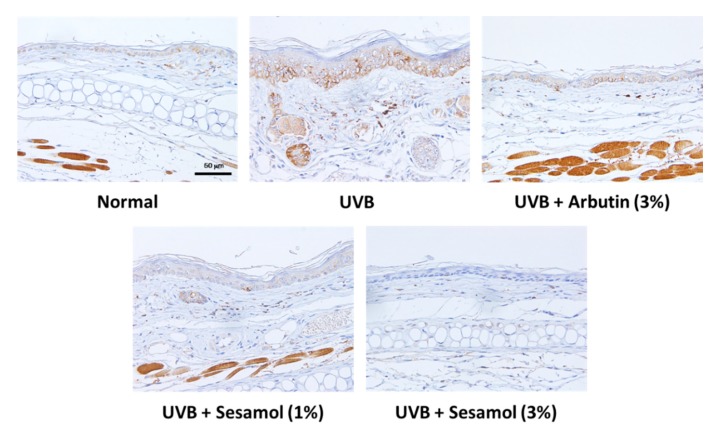

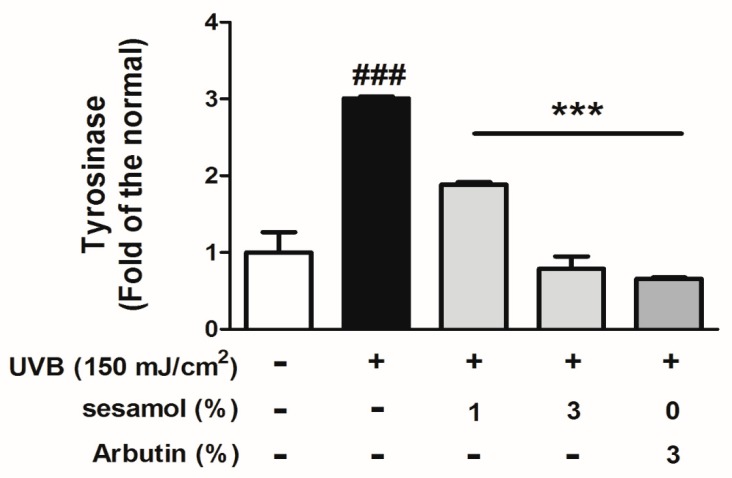
Light micrographs of histological sections stained with antibodies against tyrosinase in UVB-irradiated C57BL/6 mice that received topical application of sesamol once a day to the ear skin. The tyrosinase level increased in the UVB-irradiated group, whereas topical application of sesamol reduced the effect. (Significant difference versus control: ###, *p* < 0.001. Significant difference versus UVB treated group: ***, *p* < 0.001.).

After UVB irradiation exposure, the tyrosinase level in the epidermis of the mouse skin increased. However, as depicted in Figure 9, sesamol treatment reduced tyrosinase expression in a dose-dependent manner.

**Figure 10:**
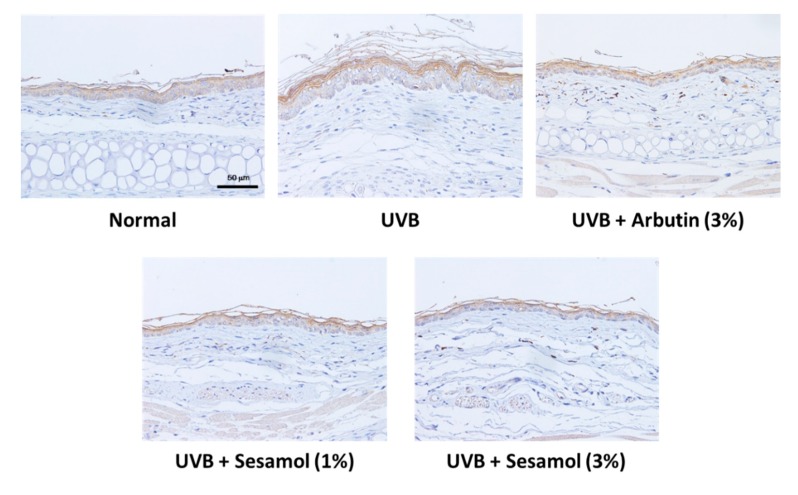

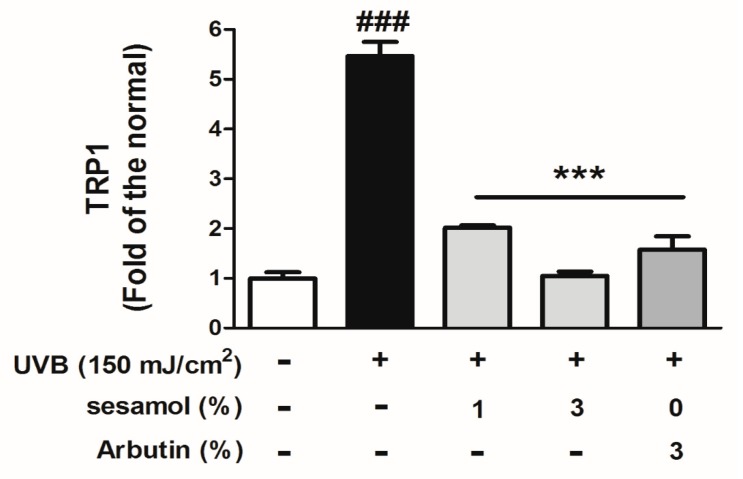
Light micrographs of histological sections stained with tyrosinase-related protein-1 (TRP-1) in UVB-irradiated C57BL/6 mice that received topical application of sesamol once a day to the ear skin. The TRP-1 level increased in the UVB-irradiated group, whereas topical application of sesamol reduced the effect. (Significant difference versus control: ###, *p* < 0.001. Significant difference versus UVB treated group: ***, *p* < 0.001.).

**Figure 11:**
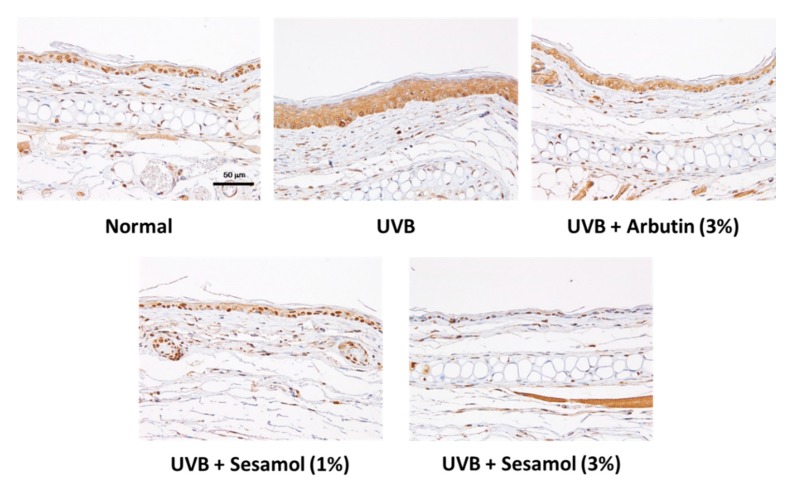

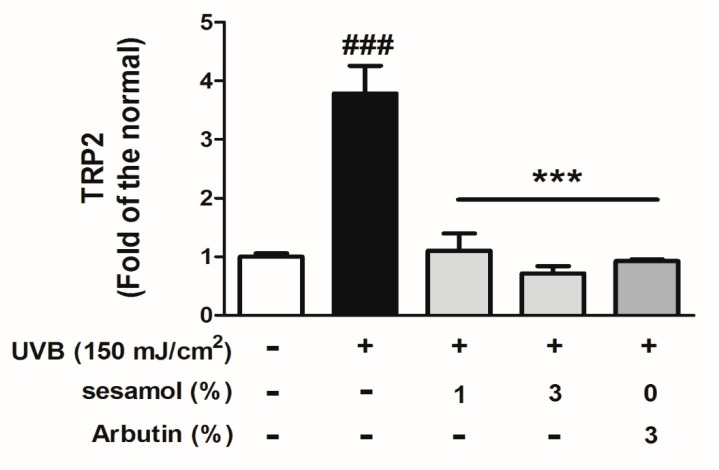
Light micrographs of histological sections stained with dopachrome tautomerase (TRP-2) in UVB-irradiated C57BL/6 mice that received topical application of sesamol once a day to the ear skin. The TRP-2 level increased in the UVB-irradiated group, whereas topical application of sesamol reduced the effect. (Significant difference versus control: ###, *p* < 0.001. Significant difference versus UVB treated group: ***, *p* < 0.001.).

**Figure 12:**
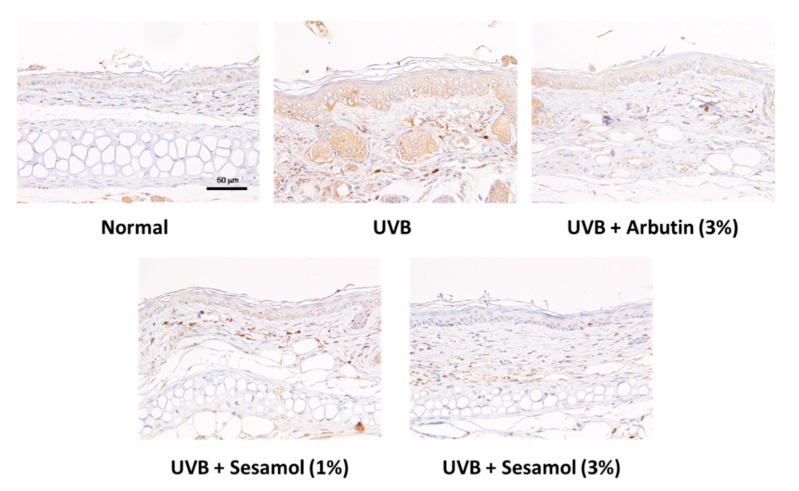

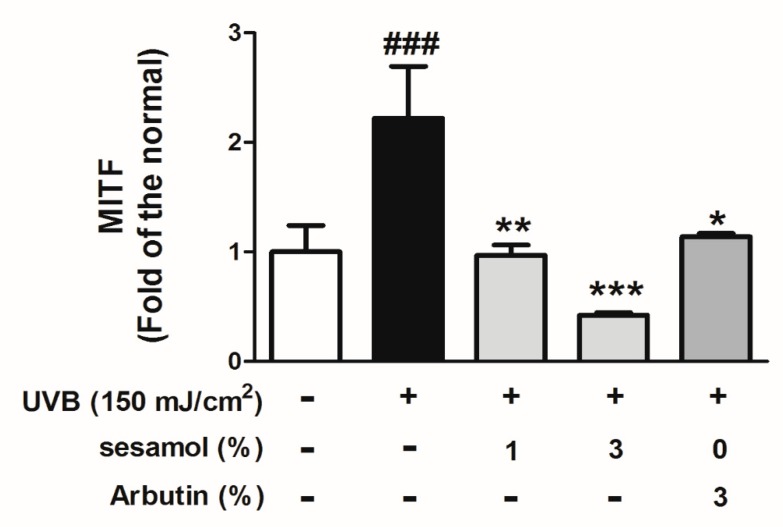
Light micrographs of histological sections stained with microphthalmia-associated transcription factor (MITF) in UVB-irradiated C57BL/6 mice that received topical application of sesamol once a day to the ear skin. (Significant difference versus control: ###, *p* < 0.001. Significant difference versus UVB treated group: *, *p* < 0.05, **, *p* < 0.01, ***, *p* < 0.001.).

UVB irradiation stimulates melanin-synthesis-related protein expression and induces melanogenesis. Figure 10, Figure 11 and Figure 12 illustrate that UVB-irradiation-induced TRP-1, TRP-2, and MITF expression in the epidermis of the mouse skin, whereas the topical application of sesamol significantly inhibited UVB-induced melanin-synthesis-related protein expression, which inhibited pigmentation in the mouse skin.

## 4. Discussion

Exposure to UVB irradiation is the major risk factor for skin cancer and skin photoaging. UV irradiation induces oxidative DNA damage through the generation of ROS, resulting in skin cancer [28]. Agents or materials with antioxidative activity may protect the skin from UV irradiation damage by quenching free radicals [29,30]. Sesame and sesamol was reported with antioxidative and free radical scavenging activities [17,18,20,31,32]. Sesamol increased the activities of superoxide dismutase, catalase, glutathione peroxidase, and glutathione, which were reduced by UVB irradiation [20]. In addition, sesamol inhibited UVB irradiation-induced cytotoxicity, intracellular ROS, lipid peroxidation, and oxidative DNA damage in human skin fibroblasts [20]. Sesamol, which contains a phenol group, can scavenge ROS and free radicals and is a strong antioxidant that can inhibit monophenolase and diphenolase activity [20]. UV irradiation exposure also induces hyperplasia of the epidermis and collagen degradation in the dermis [33,34]. However, topical application of agents or materials with antioxidative activity ameliorates the injury caused by UVB. In this study, sesamol reduced UVB-induced skin damage. Melanogenesis is a series of oxidation reactions; materials with antioxidative activity may inhibit the process of melanin synthesis [8,13,35]. The free radicals scavenging activity of sesamol may contribute to its anti-melanogenesis activity.

Melanin is synthesized by several oxidative reactions that are initiated by tyrosine and multiple enzymes [1]. Tyrosinase is the rate-limiting enzyme in melanin synthesis [35]. Tyrosinase determines the skin and hair color of mammals. Tyrosinase accumulation may cause dermatological disorders such as melasma and age spots; therefore, inhibition of tyrosinase may contribute to depigmentation. It was reported that sesamol inhibited tyrosinase activity [21,36]. Sesamol potently inhibited melanin biosynthesis in melanocytes in zebrafish [21]. In our previous study, sesamol inhibited intracellular activity and protein expression of tyrosinase in B16F10 cells [22]. Sesamol was a competitive inhibitor of diphenolase activity and a noncompetitive inhibitor of monophenolase activity in B16F10 cells [36]. The present study further demonstrated the inhibitory effect of sesamol on melanin content and tyrosinase expression in C57BL/6 mice.

In addition to tyrosinase, TRPs such as TRP-1 and TRP-2 are involved in melanin synthesis. Tyrosinase hydroxylates tyrosine to dihydroxyphenylalanine (DOPA) and oxidizes DOPA to dopaquinone [37]. TRP-2 catalyzes the conversion of dopachrome to 5,6-dihydroxyindole-2-carboxylic acid (DHICA), and TRP-1 oxidizes the conversion of DHICA to indole-5,6-quinone carboxylic acid [3]. Our previous study reported that sesamol inhibited α-MSH-induced melanogenesis in a dose-dependent manner [22]. The inhibition of TRP-1 and tyrosinase protein expressions of sesamol reduced melanin synthesis in B16F10 cells [22]. The results of immunohistochemical staining in the present study demonstrated that sesamol inhibited UVB-irradiation-induced tyrosinase, TRP-1, and TRP-2 expression in epidermis of the mouse skin. Stimulation of MC1R by α-MSH activates adenyl cyclase to increase cAMP production. MITF, a basic helix-loop-helix leucine zipper transcription factor involved in the development of melanocytes, is a major regulator of the synthesis of TRPs. MITF is the major transcription factor that plays an important role in melanogenesis. When a specific sequence of the tyrosinase gene is targeted by MITF, tyrosinase expression is upregulated. The results of immunohistochemical staining in the present study demonstrated that sesamol inhibited UVB-irradiation-induced MITF expression in epidermis of the mouse skin. These data were found to be in agreement with our previous studies regarding inhibition of intracellular MITF in B16F10 cells by sesamol. Future experiments using western blotting or other assays would be necessary to ensure the results of immunohistochemical staining.

Keratinocytes secrete hormones and growth factors that bind to specific receptors on the membrane of melanocytes, which activate downstream signal transduction to induce melanin synthesis and melanosome maturation [3]. Our previous study demonstrated that sesamol inhibited α-MSH-induced MC1R and MITF expression, which reduced melanin synthesis in B16F10 cells [22]. Sesamol also inhibited melanin biosynthesis in melan-a cell through inhibiting tyrosinase and MITF expression [21]. Sesamol also inhibited tyrosinase and melanin in zebrafish [21]. In this study, sesamol inhibited UVB-induced expression of tyrosinase, TRP-1, TRP-2, and MITF, resulting in the decrease of hyperpigmentation in the mice. The results of sesamol in C567BL/6 mice are consistent with those of sesamol in B16F10 cells. 

## 5. Conclusions

This study demonstrated the antimelanogenic activity of sesamol in C57BL/6 mice. Sesamol reduced the melanin index and elevated the brightness of the mouse skin. Sesamol exhibited antimelanogenic activity by inhibiting MITF, tyrosinase, TRP-2, and TRP-1 expression in the mouse skin. In conclusion, sesamol may be used in skin whitening products in the future.

## Abbreviations

α-MSH	α-melanocyte-stimulating hormone
AKT	protein kinase B
ERK	extracellular signal-regulated kinases
MC1R	melanocortin 1 receptor
MEK	mitogen-activated protein kinase kinase
MITF	microphthalmia-associated transcription factor
PEG	polyethylene glycol
TRP-1	tyrosinase-related protein-1
TRP-2	tyrosinase-related protein-2;
UV	ultraviolet
